# Characterization and identification of the powdery mildew resistance gene in wheat breeding line ShiCG15–009

**DOI:** 10.1186/s12870-023-04132-y

**Published:** 2023-02-23

**Authors:** Wenjing Zhang, Ziyang Yu, Dongmei Wang, Luning Xiao, Fuyu Su, Yanjun Mu, Jianpeng Zheng, Linzhi Li, Yan Yin, Tianying Yu, Yuli Jin, Pengtao Ma

**Affiliations:** 1 College of Life Sciences, Yantai University, Yantai, 264005 China; 2 Institute of Grain and Oil Crops, Yantai Academy of Agricultural Sciences, Yantai, 265500 China

**Keywords:** *Triticum aestivum* L., Powdery mildew, Molecular mapping, *PmCG15–009*, MAS

## Abstract

Powdery mildew, caused by *Blumeria graminis* f. sp. *tritici* (*Bgt*), is a serious fungal disease that critically threatens the yield and quality of wheat. Utilization of host resistance is the most effective and economical method to control this disease. In our study, a wheat breeding line ShiCG15–009, released from Hebei Province, was highly resistant to powdery mildew at all stages. To dissect its genetic basis, ShiCG15–009 was crossed with the susceptible cultivar Yannong 21 to produce F_1_, F_2_ and F_2:3_ progenies. After genetic analysis, a single dominant gene, tentatively designated *PmCG15–009*, was proved to confer resistance to *Bgt* isolate E09. Further molecular markers analysis showed that *PmCG15–009* was located on chromosome 2BL and flanked by markers *XCINAU130* and *XCINAU143* with the genetic distances 0.2 and 0.4 cM, respectively, corresponding to a physic interval of 705.14–723.48 Mb referred to the Chinese Spring reference genome sequence v2.1. *PmCG15–009* was most likely a new gene differed from the documented *Pm* genes on chromosome 2BL since its different origin, genetic diversity, and physical position. To analyze and identify the candidate genes, six genes associated with disease resistance in the candidate interval were confirmed to be associated with *PmCG15–009* via qRT-PCR analysis using the parents ShiCG15–009 and Yannong 21 and time-course analysis post-inoculation with *Bgt* isolate E09. To accelerate the transfer of *PmCG15–009* using marker-assisted selection (MAS), 18 closely or co-segregated markers were evaluated and confirmed to be suitable for tracing *PmCG15–009*, when it was transferred into different wheat cultivars.

**Supplementary Information:**

The online version contains supplementary material available at 10.1186/s12870-023-04132-y.

## Background

Common wheat (*Triticum aestivum* L., 2n = 6x = 42, AABBDD) is the most widely grown cereal crop throughout the world, which provides approximately 20% of calories for humans [1]. However, the yield and quality of wheat are affected by multiple pathogens. Powdery mildew, caused by *Blumeria graminis* f. sp. *tritici* (*Bgt*), is one of the most common diseases of wheat, with the potential to cause up to 40% grain loss or even worse during severe epidemics [2, 3]. Therefore, it’s significantly important to control the occurrence of powdery mildew. Although chemical and agricultural treatments are the mostly used methods for disease control, resistant cultivars are preferred because of high-efficiency and environmental-friendly therefore their breeding is one of objectives that breeders pursue.

Up to now, 68 formally designated powdery mildew resistance genes at 63 loci (*Pm1*-*Pm68*, *Pm8* = *Pm17*, *Pm18* = *Pm1c*, *Pm22 = Pm1e*, *Pm23 = Pm4c*, *Pm31 = Pm21*) have been reported [4, 5]. Most of these genes are race-specific, which is easy to lose resistance with the large-scale deployment in production due to evolution of the pathogen. Although a number of resistance genes have been identified in wheat and its relatives [6], new *Bgt* isolates continue to emerge to defeat deployed *Pm* genes. Recent studies indicate that *Pm2*, *Pm3a*, *Pm3b*, *Pm3f*, *Pm4a*, *Pm6*, *Pm8*, and *Pm17* have been overcome in part or all of the USA, while *Pm1a*, *Pm3a*, and *Pm8* were defeated in Australia, China, and Egypt [7, 8]. Therefore, it is necessary to continuously search for new *Pm* genes from various resistance sources to reply to the constantly evolved *Bgt* isolates.

*Pm* genes currently reported are derived from common wheat or its relatives, including *Aegilops squarrosa*, *Ae. speltoides, Ae. longissima, Ae. ovata*, *Dasypyrum villosum*, *T. urartu, T. turgidum* var*. dicoccoides*, *T. turgidum var. dicoccum, T. turgidum* var*. durum, T. timopheevii*, *T. monococcum*, *Thinopyrum intermedium*, and rye (*Secale cereale* L.) (http://wheat.pw.usda.gov/). Generally, the genes derived from wild relatives of wheat cannot be directly applied in wheat production due to the poor agronomic traits or other undesirable linkage drag, such as *Pm6* derived from *T. timopheevii* and *Pm8* from rye [9, 10], have been widely used in wheat powdery mildew resistance improvement. However, it is a great challenge to eliminate linkage drag associated with alien genes. In fact, nearly half of the reported *Pm* genes are derived from common wheat, such as *Pm52* [11], *Pm59* [12] and *Pm65* [13]*.* These genes could be directly applied to breeding practices through conventional cross and backcross ways. Therefore, mining and utilizing novel genes/alleles from common wheat is more attractive to balance resistance and applicability.

Once the novel disease-resistance gene(s) is identified, its accurate and efficient transfer or pyramiding is important in breeding programs. Marker-assisted selection (MAS) based on the gene-linked DNA markers matching the target phenotype is routinely used in the selection of desired characteristics, which is more effective than conventional breeding because it can accelerate the breeding process [14]. In view of this technology, reliable markers are the key factor. So far, although numerous molecular markers related to *Pm* genes have been developed and identified, most of them are commonly used for gene mapping or cloning, and their effectiveness in different genetic backgrounds needs to be further verified.

Wheat breeding line ShiCG15–009, released from Hebei Province, showed high resistance at the seedling and adult stages to powdery mildew and elite agronomic traits for consecutive years. In the present study, to better clarify and use the powdery mildew resistance in ShiCG15–009, the objectives of this study were to (i) characterize the powdery mildew resistance gene(s) and determine its inheritance; (ii) rapidly map the *Pm* gene(s); (iii) predict and analyze the candidate genes in the targeted interval; (iv) evaluate and develop the tightly linked or co-segregated markers suitable for MAS.

## Results

### Inheritance of powdery mildew resistance in ShiCG15–009

**Fig. 1:**
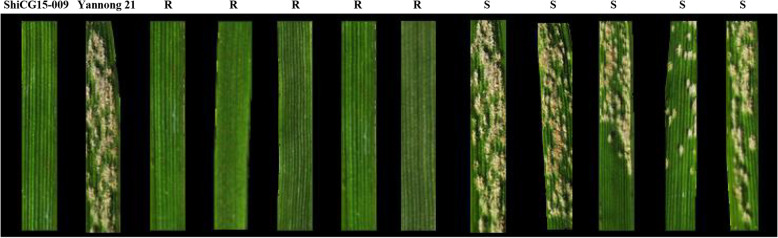
The phenotype of resistant parent ShiCG15–009, susceptible parent Yannong 21, and part of F_2_ plants about 14 days after inoculation with powdery mildew *Blumeria graminis* f. sp. *tritici* (*Bgt*) isolate E09

When inoculated with isolate E09, ShiCG15–009 was highly resistant with IT 0, whereas Yannong 21 was highly susceptible with IT 4 (Fig. 1; Table 1). All the 10 F_1_ seedlings of the cross ShiCG15–009 × Yannong 21 were resistant with ITs 0–1, indicating the resistance of ShiCG15–009 to *Bgt* isolate E09 was controlled by dominant *Pm* gene(s). Among 115 F_2_ plants, 79 were resistant with ITs 0–2 and 36 were susceptible with ITs 3–4, fitting a 3:1 ratio (χ^2^ = 2.11, *P* = 0.15). Subsequently, all 115 F_2_ plants were transplanted in the field to generate F_2:3_ families for the confirmation of the homozygous or heterozygous genotype of the resistant F_2_ plants. Twenty plants of each F_2:3_ family were evaluated for powdery mildew response. The ratios of homozygous resistant (RR): segregating (Rr): homozygous susceptible (rr) families from the cross ShiCG15–009 × Yannong 21 were consistent with the expected 1:2:1 (*χ*^*2*^ = 3.42; *P* = 0.18) (Fig. 1; Table 1). Therefore, we concluded that the resistance to *Bgt* isolate E09 in ShiCG15–009 was controlled by a single dominant gene, tentatively designated as *PmCG15–009*. More importantly, wheat line ShiCG15–009 was also resistant to the highly virulent isolates E20 and E31 with IT 0 and IT1, respectively (Table S1).

**Table 1:** Genetic analysis of resistance to *Blumeria graminis* f. sp. *tritici* (*Bgt*) isolate E09 in F_1,_ F_2_ and F_2:3_ population from cross ShiCG15–009 and susceptible parent Yannong 21

Parent and Cross	Generation	Observed ratio			Expected ratio	***χ***^**2a**^	***P***
		HR	Seg	HS			
ShiCG15–009	P_R_	10					
Yannong 21	P_S_			10			
ShiCG15–009 × Yannong 21	F_1_	10					
ShiCG15–009 × Yannong 21	F_2_	79		36	3:1	2.11	0.15
ShiCG15–009 × Yannong 21	F_2:3_	22	57	36	1:2:1	3.42	0.18

*P*_*R*_ Resistant parent, *Ps* Susceptible parent, *HR* Homozygous resistant, *Seg* Segregating, *HS* Homozygous susceptible

^a^Values for significance at *P* = 0.05 are 3.84 (*df* = 1) and 5.99 (*df* = 2)

### Molecular mapping of *PmCG15–009*

**Fig. 2:**
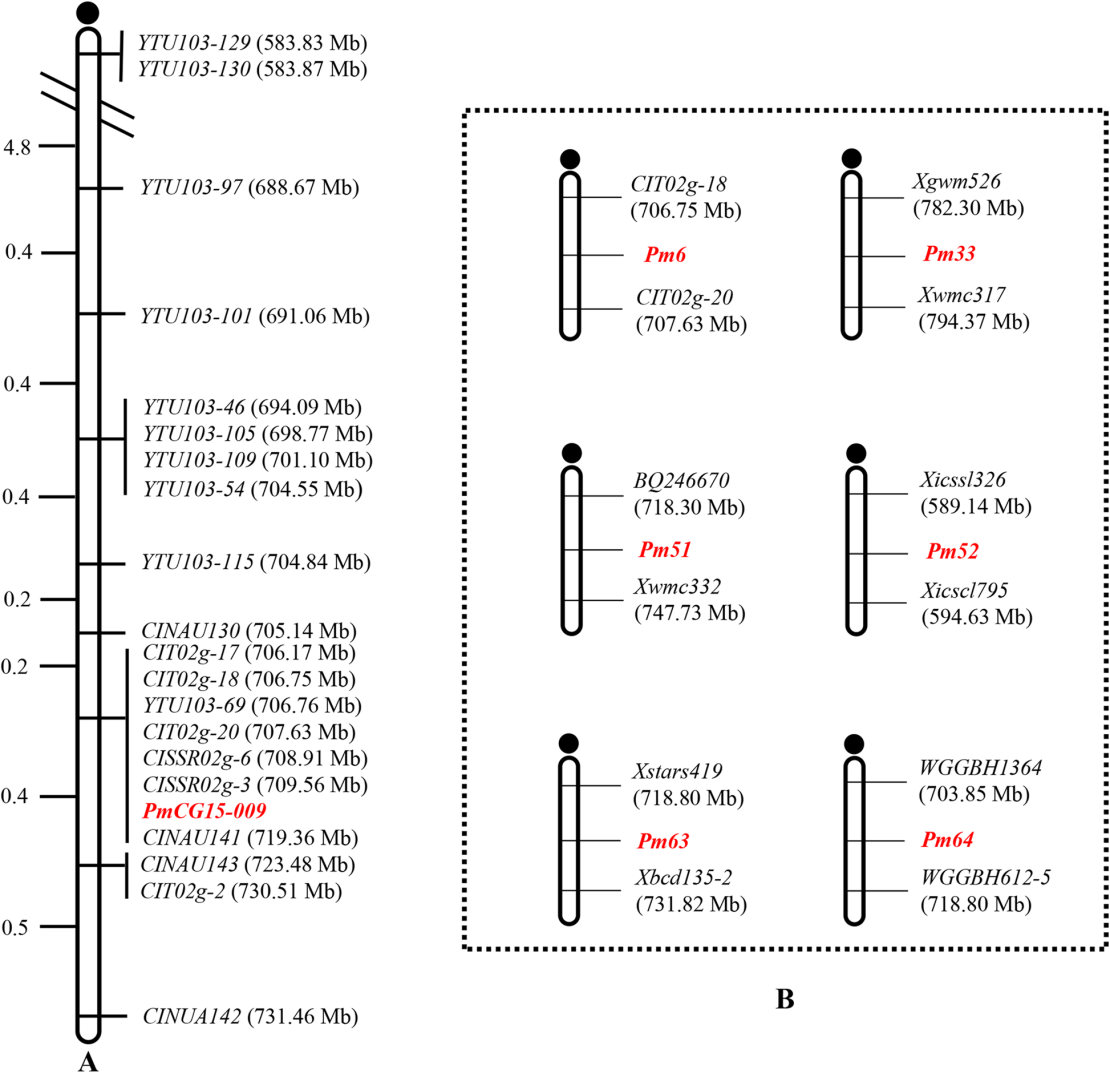
Linkage map of *PmCG15–009* using the F_2:3_ families of ShiCG15–009 × Yannong 21 (**A**) and the physical intervals of documented formally designated powdery mildew resistance genes on chromosome arm 2BL (**B**). Genetic distances in cM are showed to the left. The black filled circle represents the centromere

**Fig. 3:**
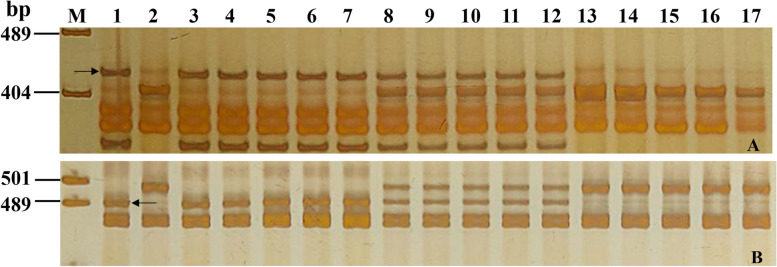
Amplification patterns of *PmCG15–009*-linked markers *YTU103–101* (**A**) and *CIT02g–17* (**B**) in genotyping resistant parent ShiCG15–009, susceptible parent Yannong 21, and randomly selected F_2:3_ families of ShiCG15–009 × Yannong 21. Lane M: pUC19/*Msp*I; 1: ShiCG15–009; 2: Yannong 21; 3–7: homozygous resistant F_2:3_ families; 8–12: heterozygous F_2:3_ families; 13–17: homozygous susceptible F_2:3_ families. The black arrows were used to indicate the polymorphic bands linked to *PmCG15–009*

In an initial survey of polymorphism between ShiCG15–009 and Yannong 21 and two DNA bulks with 321 molecular markers distributed across the wheat genome only ten markers which were located on chromosome 2BL amplified consistent polymorphisms between the parents and bulks. Then, these ten markers were genotyped on the entire 115 F_2:3_ families to map *PmCG15–009*. To further narrow the mapping interval, based on the Chinese Spring reference genome sequence v2.1 in the targeted region, ten developed SSR markers showed identical polymorphisms between the two parents and two DNA bulks and were also used to genotype the F_2:3_ families. Finally, *PmCG15–009* was flanked by the markers *CINAU130* and *CINAU143/CIT02g-2* with genetic distances of 0.2 cM and 0.4 cM, respectively, corresponding to 705.14–723.48 Mb physic interval according to the IWGSC Chinese Spring reference genome v2.1 (Figs. 2 and 3, Table 2).

**Table 2:** Polymorphic and linkage analysis of the markers linked to the powdery mildew resistance genes located on chromosome arm 2BL using the mapping population derived from the cross of ShiCG15–009 × Yannong 21

Marker	Resistance genes	Polymorphism		Linkage to ***PmCG15–009***	Forward primer (5′–3′)	Reverse primer (5′–3′)	Cultivars	References
		Parents	F_**2:3**_ bulks					
*CIT02g-1*	*Pm6*	–	–	–	TGTCACCTACCCATTCAGCT	TTCTCCAATGCTTCGAGTGC	Coker747	[15]
*CIT02g-2*	*Pm6*	+	+	+	GAGAGCATTCGTCGGTTTCC	ATTCGACCGCCTCAAATCCA		
*CIT02g-3*	*Pm6*	–	–	–	GACCGTGCCTTCCATTGTTG	TGTTCACACAAGCAGCAAGT		
*CIT02g-4*	*Pm6*	–	–	–	TGACCCTAAAACAGTCTCAAAGA	TGTTGTAAATGAGAAGTGCACCT		
*CIT02g-5*	*Pm6*	–	–	–	GGTCACCTTCTTCATAGCGC	GGTCACCTTCTTCATAGCGC		
*CIT02g-6*	*Pm6*	–	–	–	CGGCATCGTCCAGGAAATG	TGCTTTGGTTCGAGTTGGTG		
*CIT02g-7*	*Pm6*	–	–	–	CCTCTCTTCCTGTCCCTTATGG	ACTACCGATGAGAGTTCCAGA		
*CIT02g-8*	*Pm6*	–	–	–	AAGAAAGCGCGCACCATG	GCAGTCCACGAACCGCTC		
*CIT02g-9*	*Pm6*	–	–	–	AAATCGAAGCCTTGCACCAA	GGACAAAGTGCGCGAAGT		
*CIT02g-10*	*Pm6*	–	–	–	TGGGACTGGTTAGCACTTGA	CGATGAGGAATAAGTGGGCA		
*CIT02g-11*	*Pm6*	–	–	–	CAAAGCTTGCAAGATGGGTG	TTCCAGCCCCTCTAGTGATC		
*CIT02g-12*	*Pm6*	–	–	–	TGGAACGTCTAGACCACAGG	TGGAACGTCTAGACCACAGG		
*CIT02g-13*	*Pm6*	–	–	–	AGAGAAGTGGAGGTGATGGC	CACGGAGGCTGGGTTCAC		
*CIT02g-14*	*Pm6*	–	–	–	TCTTCCTCTCTTCCTGTCCC	ACTACCGATGAGAGTTCCAGA		
*CIT02g-15*	*Pm6*	–	–	–	GAGAGCATTCGTCGGTTTCC	GCTTCCTGGATCATCTGAGC		
*CIT02g-16*	*Pm6*	–	–	–	GCATCAATAAATCCCTTTCTGCA	TTTCCTCCAGTTCATCGCCC		
*CIT02g-17*	*Pm6*	+	+	+	CTGGATGAACTTCCCCAAAA	TCAATCTTGAACATCTCCCTCA		
*CIT02g-18*	*Pm6*	+	+	+	GGCCTTAGTGGTGATGCAGT	GCGGCTTGTCGGTGTATAG		
*CIT02g-19*	*Pm6*	–	–	–	TCGTTCACACTCAACTCCCA	AGCGAGATCCCATGACTGAC		
*CIT02g-20*	*Pm6*	+	+	+	CGTGCCTTCCATTGTTGTAT	TGTTCACACAAGCAGCAAGTT		
*CIT02g-21*	*Pm6*	–	–	–	TTTGGGCCTGCGACGATC	ACGGTGTTATTCCTAGCATGC		
*CIT02g-22*	*Pm6*	–	–	–	CTCTACGAGCTGTCTTCGCT	TCCCTTGGTAGTACTTGGACA		
*CISSR02g-1*	*Pm6*	–	–	–	TGTCATTTACTCGTGTGCTTCA	CCTTACGCTTTCCTCATAAACC		
*CISSR02g-2*	*Pm6*	–	–	–	GACTACAACTACCTTCCCGTGG	AGGATGAAAACCTCGACACACT		
*CISSR02g-3*	*Pm6*	+	+	+	CTAAACCATAAGCAATCCCCTG	GTCTACAACTACCTTCCCGTGG		
*CISSR02g-4*	*Pm6*	–	–	–	TTCGTAGGTTTTGTGCATGTTC	AGTTAGGGTAGGAAGAGGTGGG		
*CISSR02g-5*	*Pm6*	–	–	–	ACTTCCAGCAAATGTTGTAGCC	GTCGAGAGTTGAGGGTCGTC		
*CISSR02g-6*	*Pm6*	+	+	+	TAAGCAACATCTCATCCCCTTT	GAATACGCCTCCACTCATACCT		
*CINAU117*	*Pm6*	–	–	–	GACCCAAGAGGCGTTGATTA	CATGTGTGCCAAATTCAAGC		[16]
*CINAU118*	*Pm6*	–	–	–	GCTGTGACTGCTGGATTCAA	ACCGGGACTGTGTAGACTGG		
*CINAU119*	*Pm6*	–	–	–	CTTCGTTGCTCGAAAGGTTC	CGGGTGAAACATCTTCTGGT		
*CINAU120*	*Pm6*	–	–	–	GCCATGGCTAAGGAAGAAGA	ACCTTGGCGAGCTTCTTGAC		
*CINAU121*	*Pm6*	–	–	–	CCTAGACTGGCCAAGACGAT	ATGGTTTGATTCACCAGCAA		
*CINAU122*	*Pm6*	–	–	–	CACCTACCTCGTCAACGG	GAGTGCTCCACTGTAAAGCC		
*CINAU123*	*Pm6*	–	–	–	TTGTACGCCATCGACACATT	CCGAACAGAGTTTTGCCTTC		
*CINAU124*	*Pm6*	–	–	–	GAGTGCTCCACTGTAAAGCC	CACCTTTGTAGACAGTCCCG		
*CINAU125*	*Pm6*	–	–	–	CCTCTTCCTGACCATCTTCC	TGACAGTCACTCCAATCACG		
*CINAU126*	*Pm6*	–	–	–	TCATTTGGTTGCATAGTTGC	AATTTAGCAGTATTCTTAGCTTCCC		
*CINAU127*	*Pm6*	–	–	–	AATTTAGCAGTATTCTTAGCTTCCC	ATGGGCCGTACAAGAAAGTG		
*CINAU128*	*Pm6*	–	–	–	TCGAACATGGCTGTGATGAT	GGCTCAGCTTTACCAAGAGC		
*CINAU129*	*Pm6*	–	–	–	ATCTTGCAGCTTTTGCGTTT	GCTCCCTGACACTCTTGAGG		
*CINAU130*	*Pm6*	+	+	+	GGCGAGAAAATGTTGTCCAT	AGAAGAGCTGGAGCACCTTG		
*CINAU131*	*Pm6*	–	–	–	CAACTGCTGGCTCTTCTTCC	GGAACAGCAGCGTCTTCTTC		
*CINAU132*	*Pm6*	–	–	–	GTGGCTACACCCAAACGG	CAGATCAACGGGAGACATCAC		
*CINAU133*	*Pm6*	–	–	–	AAGAACCATATCTGGGCTGTC	TACAACAAGATGCCGCAGGCTAACA		
*CINAU134*	*Pm6*	–	–	–	ATCAACAAGATCTTCGACGG	CTTTGTCTGAACATTGCTGC		
*CINAU135*	*Pm6*	–	–	–	TTGGTGACGCAGTAATGGAA	TGTGACAGAGCTAGGGCAAG		
*CINAU136*	*Pm6*	–	–	–	CTGACTGCGCCTTATGTTGA	CCGTGGCTTGATGGAGTCATA		
*CINAU137*	*Pm6*	–	–	–	GGACAATGAGAAAGCAAAGG	CTTTGCAAGAGCATCAGAGG		
*CINAU138*	*Pm6*	–	–	–	TTCCCGAAGGACTACCATTG	TCCAGTCACCTCTGGAGCTT		
*CINAU139*	*Pm6*	–	–	–	CAAAGGAGCCTTTCGATGAG	GGATTCGGGTAGCTTGCATA		
*CINAU140*	*Pm6*	–	–	–	CACGGTGGAAGTCACTAACC	CAGTTTCCAAGGCATAGGG		
*CINAU141*	*Pm6*	+	+	+	CACACATGGCAAGTTACAGG	ATCAGACTTGCTTGCTCACC		
*CINAU142*	*Pm6*	+	+	+	CGACTACGTGACGCTCAAGA	ACTTGTCGTCGAGGAGGATG		
*CINAU143*	*Pm6*	+	+	+	GTTGGTGGTTGAAAAGATGG	AGTATGCACCTTCGATTTGC		
*CINAU144*	*Pm6*	–	–	–	GCTCCTCAGCAAATGCCTAC	GATGAAGTGGTGAGCAAGCA		
*NAU/STS*_*BCD135–2*_	*Pm6*	–	–	–	GCTCCGAAGCAAGAGAAGAA	TCTGCTGGTCCTCTGATGTG		[17]
*Xwmc317*	*Pm33*	–	–	–	TGCTAGCAATGCTCCGGGTAAC	TCACGAAACCTTTTCCTCCTCC	Am9/3	[18]
*Xgwm526*	*Pm33*	–	–	–	CAATAGTTCTGTGAGAGCTGCG	CCAACCCAAATACACATTCTCA		
*BQ246670*	*Pm51*	–	–	–	ACATGAGTGAGTTGTGAGTC	AGAAGGCACACTGCTGGAAC	CH7086	[19]
*BE444894*	*Pm51*	–	–	–	CAATGGGGGTCTTATGGATG	GATGTTGCAGACGGGGTAGT		
*BE405017*	*Pm51*	–	–	–	CTTACTGGTGGACATGGGCT	CGCAGGGCTATCTTGTTCTC		
*Xbarc159*	*Pm51*	–	–	–	CGCAATTTATTATCGGTTTTAGGAA	CGCCCGATAGTTTTTCTAATTTCTGA		
*Xwmc332*	*Pm51*	–	–	–	CATTTACAAAGCGCATGAAGCC	GAAAACTTTGGGAACAAGAGCA		
*Cos66*	*Pm51*	–	–	–	CACGGTGGAAGTCACTAACC	CAGTTTCCAAGGCATAGGG		
*Xicsl34*	*Pm52*	–	–	–	GTCCAATCGATCAACTTCAG	GACTAGCTCGCTCTGGATTA	Liangxing 99	[20]
*Xicsl62*	*Pm52*	–	–	–	AGCAAAGCAATTAGGAGAGTT	CTGCGACTGTTTTCTTTTAAC		
*Xicsl90*	*Pm52*	–	–	–	AGACTGGGTGCTAGTTGTGT	TGACTTGTCACTGGTTTTCTC		
*Xicsl163*	*Pm52*	–	–	–	GAGAGTACAAAAGGCAGAGG	ACATAGGGAAATCGAATAAGG		
*Xicsl224*	*Pm52*	–	–	–	TGCTGTGCTACTTTTGCTACT	TCTCCCAATCTATCAACGTAA		
*Xicsl234*	*Pm52*	–	–	–	TCTCAGTTTTCACCTCCACTA	CCTTGCTAGAAAAAGGAGAAT		
*Xicsl275*	*Pm52*	–	–	–	CCGTCCGTATATTCAATTACTC	GCGTTTGCAAGTACAGACTAC		
*Xicsl306*	*Pm52*	–	–	–	GCGTTTGCAAGTACAGACTAC	GTAGTAAAATGGCAGCAGAGA		
*Xicscl437*	*Pm52*	–	–	–	CTGTTAGCAAGAACCATTAGG	GGAATAGCTGGAAGTCTTCTG		
*Xicscl445*	*Pm52*	–	–	–	GGAATAGCTGGAAGTCTTCTG	TAAACAACTCCATGGTTCAGT		
*Xicscl726*	*Pm52*	–	–	–	GCTGCTGAGTAGCTGTATGAG	CTATCATGGAACTTGCAAAAC		
*Xicscl795*	*Pm52*	–	–	–	GTCAACCTCATCTTCTCCTG	GTCAACCTCATCTTCTCCTG		
*Xicssl173*	*Pm52*	–	–	–	GGAAACTCAATTCATCACAAG	GGCTGAGGGTATGTACAAGTAG		
*Xicssl174*	*Pm52*	–	–	–	AACAAGCTTAACGTGTACCAA	AAAGCTTGCATGCTATAATGT		
*Xicssl326*	*Pm52*	–	–	–	AAGATGCACTTACCCAAAAAC	TGCTACATATAACTGCTGCTG		
*Xwmc175*	*Pm52*	–	–	–	GCTCAGTCAAACCGCTACTTCT	CACTACTCCAATCTATCGCCGT		[21]
*Xwmc441*	*Pm52*	–	–	–	TCCAGTAGAGCACCTTTCATT	ATCACGAAGATAAACAAACGG		
*Xgwm120*	*Pm52*	–	–	–	GATCCACCTTCCTCTCTCTC	GATTATACTGGTGCCGAAAC		
*Xbcd135–2*	*Pm63*	–	–	–	GCTCCGAAGCAAGAGAAGAA	TCTGCTGGTCCTCTGATGTG	PI 628024	[22]
*Xstars419*	*Pm63*	–	–	–	GCCCTTGTCAGTTTCAGTCC	GTCGATCGCTCCACCTCTAC		
*Xgwm120*	*Pm63*	–	–	–	GATCCACCTTCCTCTCTCTC	GATTATACTGGTGCCGAAAC		
*Xwmc175*	*Pm63*	–	–	–	GCTCAGTCAAACCGCTACTTCT	CACTACTCCAATCTATCGCCGT		
*Xwmc441*	*Pm63*	–	–	–	TCCAGTAGAGCACCTTTCATT	ATCACGAAGATAAACAAACGG		
*Xwmc332*	*Pm63*	–	–	–	CATTTACAAAGCGCATGAAGCC	GAAAACTTTGGGAACAAGAGCA		
*WGGBH1364*	*Pm64*	–	–	–	CCAAGAAATGGAGTGTTTGA	CAATTATTGGGATCAACACC	WE35	[23]
*WGGBH218*	*Pm64*	–	–	–	CCTTCCTCCGGTAACTCATA	CGAGCTAGCAATCAGAGAAG		
*WGGBH1099*	*Pm64*	–	–	–	CGAGCTAGCAATCAGAGAAG	AGGCGGTCTACTGGATTATATGT		
*WGGBH913*	*Pm64*	–	–	–	ACTGAAACGACAGCTTTTAGG	GGTGAGCTAGTTTGCTCTGTT		
*WGGBH252*	*Pm64*	–	–	–	GGTGAGCTAGTTTGCTCTGTT	GGATTGGACTATTAGTCAACG		
*WGGBH1212*	*Pm64*	–	–	–	AACCTCAGTAACCATTGCCAAG	CTCACGCCTTCAACTCATCAG		
*WGGBH612–5*	*Pm64*	–	–	–	TCTTGCCCTTGTCAGTTTCAG	TACGTGCGAGTAAGAGTAGGAG		
*WGGBH134*	*Pm64*	–	–	–	AGCTTGAATGAGGATGAAGAGT	CTTCTCTTTCTCCTTCTCCGAA		
*WGGBH686*	*Pm64*	–	–	–	CAGGGTACTGTATCAGTGTGG	AAGTGATAACACAGCTTGTCG		
*WGGBH1260*	*Pm64*	–	–	–	GACTTGCTCCTGCCTGCTA	TTCTTGGAATGTTCTGCGTGAT		
*YTU130–129*	*PmCG15–009*	+	+	+	ATCGGGAAGGCATGGTCAAG	CGAGAGGATAAGGCCGAACC	ShiCG15–0	
*YTU130–130*	*PmCG15–009*	+	+	+	GTGTACGGCAAGGTGACAGA	ATGGCAAGACTGTGGGTACG	09	
*YTU130–97*	*PmCG15–009*	+	+	+	CTAGGGCTGGACCAGTTTGG	AGTTGTGGAAATCGGCGGAT		
*YTU130–101*	*PmCG15–009*	+	+	+	GGGAGAGCCGTCAAAGAACA	CTTCTCATTTTCTCCGCGCG		
*YTU130–46*	*PmCG15–009*	+	+	+	CTTCCTCCATTGACCACGCT	GCGAGAGATTCATCCAGCGA		
*YTU130–105*	*PmCG15–009*	+	+	+	TCGAGGCGCTTCTTCACTTT	TTGCAATGGTGTTGCTCTGC		
*YTU130–109*	*PmCG15–009*	+	+	+	CCGATTACCTGCAGCTCGAT	TCCAGCTTGGACTTGTCGAC		
*YTU130–54*	*PmCG15–009*	+	+	+	AGGGCAAAAGATGGAGGTCG	TCGTTCAAGGGCATCAGCAT		
*YTU130–115*	*PmCG15–009*	+	+	+	AGGAGCTTCATGGCCTTCAC	TCACTGTGAGCGACTGACAC		
*YTU130–69*	*PmCG15–009*	+	+	+	CGAGCGTGATGTAGACCTCC	GTTTTTCCAGGCCAGCAAGG		

“+” represents polymorphic or linked, and “–” represents non-polymorphic or unlinked

### Genetic diversity comparison with the documented *pm* genes on the chromosome 2BL

To identify the relationship between *PmCG15–009* and the known formally designated *Pm* genes on chromosome 2BL, 99 closely linked or co-segregated markers, including 57 for *Pm6*, two for *Pm33*, six for *Pm51*, 18 for *Pm52*, six for *Pm63* and ten for *Pm64*, were tested the polymorphisms between the resistant and susceptible parents and bulks (Table 2). Among them, only ten markers for *Pm6* (*CINAU130*, *CIT02g-17*, *CIT02g-18*, *CIT02g-20*, *CISSR02g-6*, *CISSR02g-3*, *CINAU141*, *CINAU143*, *CIT02g-2*, *CINAU142*) amplified polymorphisms between the resistant and susceptible parents and bulks and were closely linked or co-segregated with *PmCG15–009*, while other 89 markers showed no polymorphism. Hence, *PmCG15–009* is most likely different from the known *Pm* genes on chromosome arm 2BL.

### Prediction and analysis of candidate genes

**Fig. 4:**
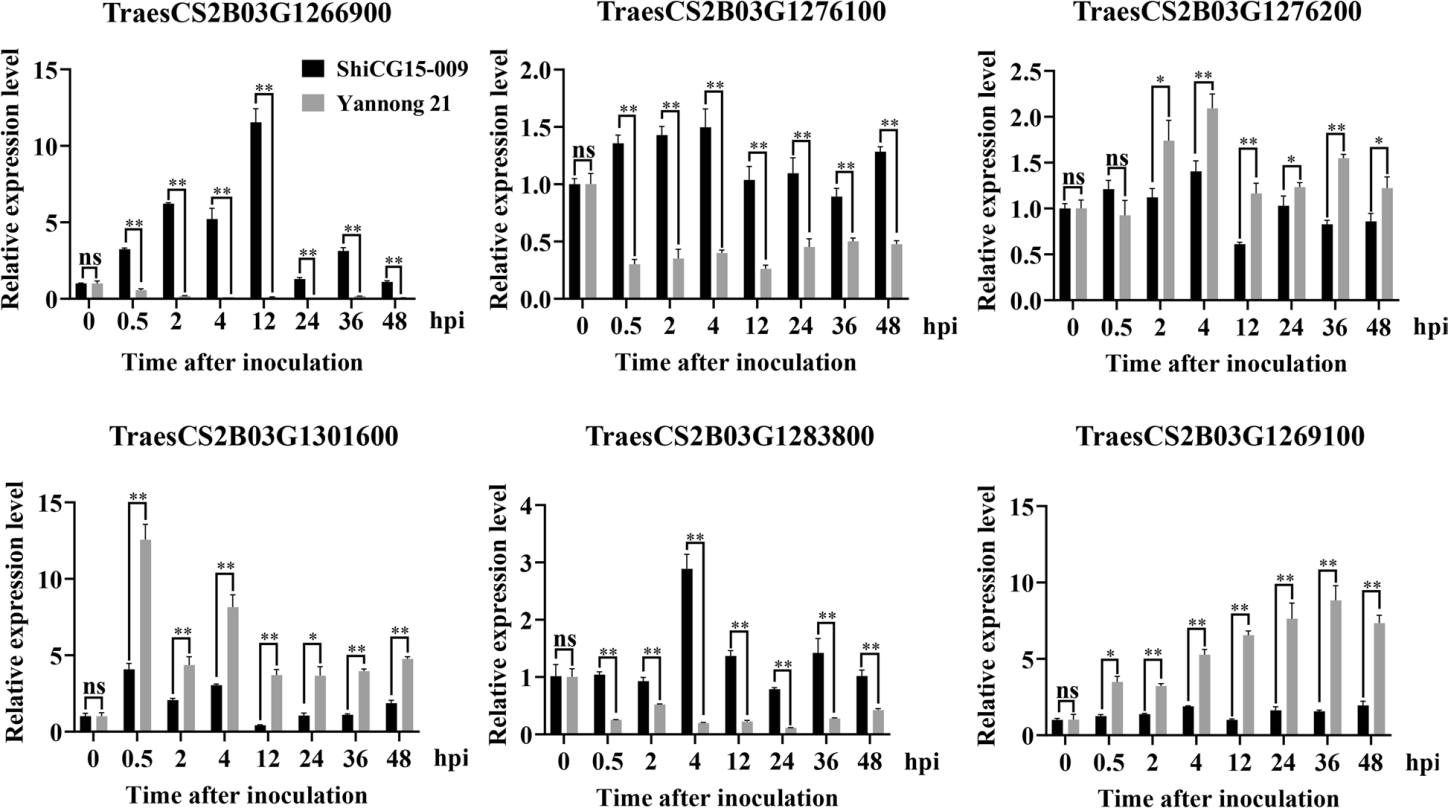
Expression pattern of *TraesCS2B03G1266900*, *TraesCS2B03G1269100*, *TraesCS2B03G1276100*, *TraesCS2B03G1276200*, *TraesCS2B03G1283800* and *TraesCS2B03G1301600* in resistant parent ShiCG15–009 and susceptible parent Yannong 21 after inoculating with *Blumeria graminis* f. sp. *tritici* (*Bgt*) isolate E09 at 0, 0.5, 2, 4, 12, 24, 36 and 48 hours post inoculation (hpi). Normalized values of target genes expression relative to *Actin* were given as mean ± SD from three replicates. Asterisks indicate significant differences (*t*-tests) between ShiCG15–009 and Yannong 21 at each time point (*P < 0.05, **P < 0.01, ns: not significant)

One hundred and ninety-four high confidence genes were annotated in the interval of 705.14–723.48 Mb on chromosome 2BL based on the IWGSC Chinese Spring reference genome v2.1. Among them, only fourteen genes are probably or supposedly associated with disease resistance, including five genes directly related to disease resistance, four genes encoding nucleotide binding site and leucine rich repeat (NBS-LRR) protein, and five genes encoding kinase (Table 3). Then, we used qRT-PCR to investigate the expression patterns of these genes in the resistant parent ShiCG15–009 and susceptible parent Yannong 21 after inoculating with *Bgt* isolate E09 at different times. As shown in Fig. 4, three genes, including *TraesCS2B03G1266900*, *TraesCS2B03G1276100* and *TraesCS2B03G1283800* were induced to express in resistant parent ShiCG15–009, whereas did not change significantly in susceptible parent Yannong 21. In contrast, *TraesCS2B03G1276200*, *TraesCS2B03G1269100* and *TraesCS2B03G1301600* were induced in the susceptible parent Yannong 21. The transcript levels of the remaining eight genes were not significantly different between ShiCG15–009 and Yannong 21. Further research is needed to identify the candidate gene for *PmCG15–009*.

**Table 3:** Gene annotation of disease-resistance related in the candidate interval of wheat powdery mildew resistance gene *PmCG15–009*

No.	Gene	Physical genomic location	Functional annotation
1	*TraesCS2B03G1266900*	chr2B:706659806..706669110	disease resistance
2	*TraesCS2B03G1269100*	chr2B:707563843..707568496	disease resistance protein
3	*TraesCS2B03G1269800*	chr2B:707673718..707677218	disease resistance
4	*TraesCS2B03G1276100*	chr2B:712330530..712334826	disease resistance
5	*TraesCS2B03G1276200*	chr2B:712406234..712410121	disease resistance
6	*TraesCS2B03G1298600*	chr2B:720465611..720467185	LRR-repeat protein
7	*TraesCS2B03G1299200*	chr2B:720513971..720515671	LRR-repeat protein
8	*TraesCS2B03G1300900*	chr2B:721231589..721233256	LRR-repeat protein
9	*TraesCS2B03G1301600*	chr2B:721291063..721292565	LRR-repeat protein
10	*TraesCS2B03G1290400*	chr2B:717184726..717191382	Protein kinase domain
11	*TraesCS2B03G1283800*	chr2B:715213560..715217715	Serine threonine-protein kinase
12	*TraesCS2B03G1284100*	chr2B:715271978..715273603	Serine threonine-protein kinase
13	*TraesCS2B03G1272100*	chr2B:709824387..709825406	cyclin-dependent protein serine/threonine kinase activity
14	*TraesCS2B03G1265500*	chr2B:706166956..706173496	Phosphatidylinositol-4-phosphate 5-kinase 9

### Molecular markers for MAS

**Fig. 5:**
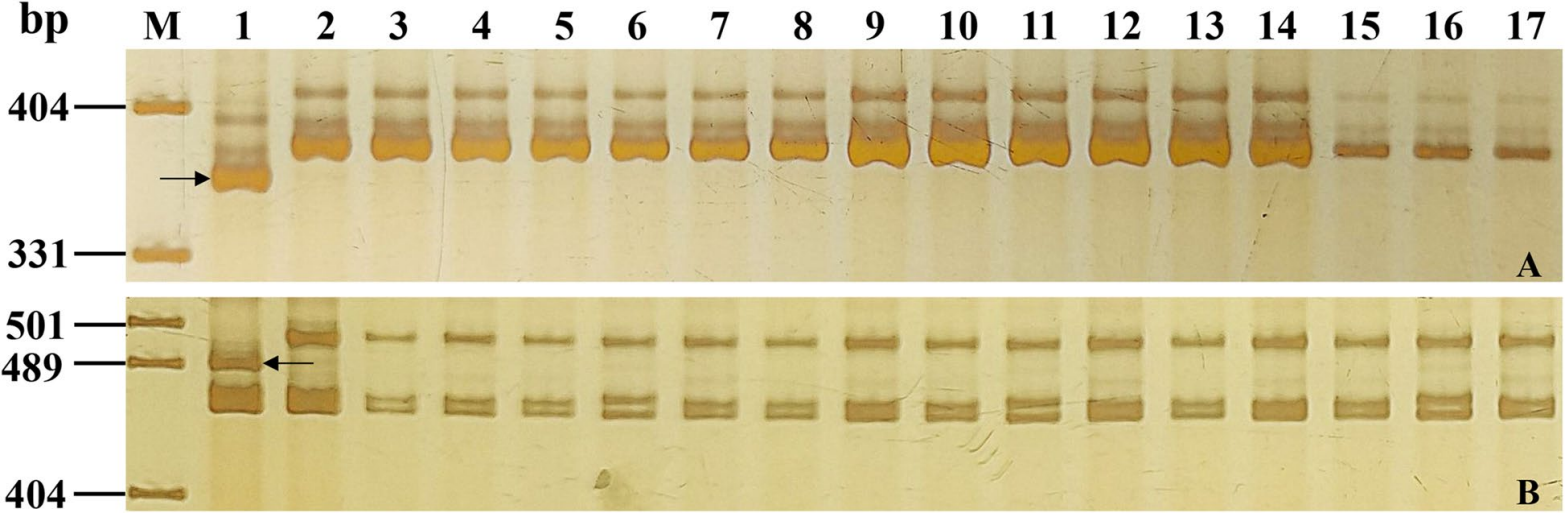
Amplification patterns of *PmCG15–009*-linked markers *CISSR02g-6* (**A**) and *CIT02g-17* (**B**) in ShiCG15–009, Yannong 21 and 15 wheat cultivars/lines susceptible to powdery mildew. M: pUC19/*Msp*I; 1: ShiCG15–009; 2: Yannong 21; 3: Shannong 1538; 4: Hanmai 13; 5: Huaimai 0226; 6: Zhoumai 27; 7: Yannong 1212; 8: Xinong 979; 9: Lumai 185; 10: Zhongyu 1311; 11: Jimai 268; 12: Tainong 1014; 13: Jimai 229; 14: Jimai 21; 15: Jimai 20; 16: Daimai 2173; 17: Zhongmai 1751. The black arrows indicate the polymorphic bands in ShiCG15–009

To better use *PmCG15–009* in MAS, 20 markers closely linked or co-segregated with *PmCG15–009* were tested for their availability in the 46 susceptible wheat cultivars/lines for MAS. Markers YTU103–130 and CIT02g-18 produced the same genotypes as ShiCG15–009 in 28 and 38 out of the 46 susceptible cultivars/lines, indicating these two markers were not informative despite closely with *PmCG15–009*. The remaining markers could amplify polymorphic bands between ShiCG15–009 and most of the 46 susceptible cultivars (Fig. 5; Table 4). These results demonstrated that these 18 markers could be used singly or in combination in MAS for tracking *PmCG15–009* when transferred into those cultivars.

**Table 4:** Validation of *PmCG15–009*-linked markers on 46 Chinese wheat cultivars/breeding lines and six reference cultivars/lines carrying known genes on the chromosome arm 2BL in marker-assisted selection (MAS) breeding

Genotypes	Region	Molecular markers																			
		1	2	3	4	5	6	7	8	9	10	11	12	13	14	15	16	17	18	19	20
16P0119	Shandong	–	–	–	–	–	–	–	–	–	–	–	+	–	–	–	–	–	–	–	–
Daimai 2173	Shandong	–	–	–	–	–	–	+	–	–	–	–	+	–	–	–	–	–	–	–	–
Hanmai 13	Hebei	–	+	+	–	–	–	–	–	–	–	–	–	–	–	–	–	–	–	–	–
Huaimai 0226	Jiangsu	–	–	+	–	–	–	–	–	–	–	–	+	–	–	–	–	–	–	–	–
Huixianhong	Shandong	+	+	+	–	–	–	–	–	–	–	–	+	–	–	–	–	–	–	–	–
Jimai 20	Shandong	–	–	–	–	+	–	–	–	–	–	–	+	–	–	–	–	–	–	–	–
Jimai 21	Shandong	–	+	–	–	–	–	–	–	–	–	–	+	–	–	–	–	–	–	–	–
Jimai 229	Shandong	–	–	–	–	–	–	+	–	–	–	–	+	–	–	–	–	–	–	–	–
Jimai 268	Shandong	+	+	–	–	–	–	–	–	–	–	–	–	–	–	–	–	–	–	–	–
Jinan 17	Shandong	–	+	+	–	–	–	–	–	–	–	–	+	–	+	–	–	–	–	–	–
Lande 677	Shandong	–	–	–	–	–	–	–	–	–	–	–	+	–	–	–	–	–	–	–	–
Liangxing 619	Shandong	–	+	–	–	–	–	–	–	–	–	–	+	–	–	–	–	–	–	–	–
Lumai 185	Shandong	–	+	+	–	–	–	–	–	–	–	–	+	–	+	–	–	–	–	–	–
Pumai 28	Henan	–	–	–	–	–	–	–	+	–	–	–	–	–	–	–	–	–	–	–	–
Qingmai 6	Shandong	–	–	–	–	–	–	–	–	–	–	–	+	–	–	–	–	–	–	–	–
Shannong 1538	Shandong	–	+	+	–	–	–	–	–	–	–	–	–	–	–	–	–	–	–	–	–
Shimai 15	Hebei	+	+	–	–	–	–	–	–	–	–	–	+	–	+	–	–	–	–	–	–
Taimai 1918	Shandong	–	–	–	–	–	–	–	–	–	–	–	+	–	–	–	–	–	–	–	–
Tainong 1014	Shandong	–	+	–	–	–	–	–	–	–	–	–	+	–	–	–	–	–	–	–	–
Womai 8	Anhui	–	+	+	–	–	–	–	–	–	–	–	–	–	+	–	–	–	–	–	–
Wunong 6	Shanxi	–	+	–	–	–	–	–	+	–	–	–	+	–	–	–	–	–	–	–	–
Xinluo 4	Henan	–	–	–	–	+	–	+	–	–	–	–	+	–	–	–	–	–	–	–	–
Xinong 979	Shanxi	–	+	+	–	–	–	–	–	–	–	–	+	–	+	–	–	–	–	–	–
Yannong 1212	Shandong	–	+	+	–	–	–	–	–	–	–	–	–	–	+	–	–	–	–	–	–
Yannong 15	Shandong	–	+	–	–	–	–	–	–	–	–	–	**+**	–	–	–	–	–	–	–	–
Yannong 161	Shandong	–	–	–	–	–	–	–	–	–	–	–	**+**	–	–	–	–	–	–	–	–
Yannong 17	Shandong	–	–	–	–	+	–	–	–	–	–	–	**+**	–	–	–	–	–	–	–	–
Yannong 191	Shandong	–	–	+	+	–	+	–	+	–	+	+	**+**	+	+	+	–	–	+	+	+
Yannong 199	Shandong	–	+	–	–	–	–	–	–	–	–	–	**+**	–	–	–	–	–	–	–	–
Yannong 215	Shandong	–	–	–	–	–	–	–	–	–	–	–	**+**	–	–	–	–	–	–	–	–
Yannong 23	Shandong	–	+	–	–	–	–	+	–	–	–	–	**+**	–	+	–	–	–	–	–	–
Yannong 24	Shandong	–	+	–	–	–	–	–	–	–	–	–	**+**	–	–	–	–	–	–	–	–
Yannong 2415	Shandong	–	+	–	+	–	–	–	–	–	–	–	**+**	–	–	–	–	–	–	–	–
Yannong 301	Shandong	–	+	–	–	–	–	+	–	–	–	–	**+**	–	–	–	–	–	–	–	–
Yannong 390	Shandong	–	+	+	–	–	–	–	–	–	–	–	**+**	–	+	–	–	–	–	–	–
Yannong 5158	Shandong	–	+	–	–	–	–	–	–	–	–	–	**+**	–	–	–	–	–	–	–	–
Yannong 572	Shandong	–	–	+	+	–	+	+	+	+	–	–	**+**	+	+	+	+	+	+	+	+
Yannong 745	Shandong	–	+	–	–	–	–	–	–	–	–	–	**+**	–	–	–	–	–	–	–	–
Yannong 836	Shandong	–	–	–	–	–	–	–	–	–	–	–	**+**	–	–	–	–	–	–	–	–
Yannong 999	Shandong	–	+	–	–	–	–	–	–	–	–	–	**+**	–	–	–	–	–	–	–	–
Zhengmai 0856	Henan	–	+	+	–	–	–	–	–	–	–	–	**+**	–	+	–	–	–	–	–	–
Zhongmai 1751	Beijing	–	–	–	–	–	–	–	–	–	–	–	–	–	–	–	–	–	–	–	–
Zhongmai 9398	Beijing	–	+	–	–	–	–	–	–	–	–	–	**+**	–	–	–	–	–	–	–	–
Zhongxinmai 77	Hebei	–	–	–	–	–	–	–	–	–	–	–	**+**	–	–	–	–	–	–	–	–
Zhongyu 1311	Beijing	–	+	–	–	–	–	–	–	–	–	–	**+**	–	–	–	–	–	–	–	–
Zhoumai 27	Henan	–	+	+	–	–	+	–	–	–	–	–	–	–	–	–	–	–	–	–	–
Coker747 (*Pm6*)	Sweden	–	–	–	–	–	–	–	–	–	+	+	+	–	+	+	+	+	+	+	+
Am9/3 (*Pm33*)	Beijing	–	–	–	–	–	–	–	–	–	–	–	–	–	–	–	–	–	–	–	–
CH7086 (*Pm51*)	Shanxi	–	–	–	–	–	–	–	–	–	–	–	–	–	–	–	–	–	–	–	–
Liangxing 99 (*Pm52*)	Hebei	–	–	–	–	–	–	–	–	–	–	–	–	–	–	–	–	–	–	–	–
PI 628024 (*Pm63*)	Iran	–	–	–	–	–	–	–	–	–	–	–	–	–	–	–	–	–	–	–	–
WE35 (*Pm64*)	Israel	–	–	–	–	–	–	–	–	–	–	–	–	–	–	–	–	–	–	–	–

1: *YTU103–129*; 2: *YTU103–130*; 3: *YTU103–97*; 4: *YTU103–101*; 5: *YTU103–46*; 6: *YTU103–105*; 7: *YTU103–109*; 8: *YTU103–54*; 9: *YTU103–115*; 10: *CINAU130*; 11: *CIT02g-17*; 12: *CIT02g-18*; 13: *YTU103–69*; 14: *CIT02g-20*; 15: *CISSR02g-6*; 16: *CISSR02g-3*; 17: *CINAU141*; 18: *CINAU143*; 19: *CIT02g-2*; 20: *CINAU142*. ‘-’ represents that the markers can’t amplify the polymorphic products that linked to *PmCG15–009* in relevant wheat cultivars/lines, and ‘+’ represents the adverse result

## Discussion

The elite wheat breeding line ShiCG15–009 shows a high level of resistance to powdery mildew at the seedling and adult stages. In this study, a dominant gene *PmCG15–009* was characterized on the long arm of chromosome 2B in ShiCG15–009, further molecular markers analysis showed that *PmCG15–009* was flanked by markers *XCINAU130* and *XCINAU143* with the genetic distances 0.2 and 0.4 cM, respectively, corresponding to a physic interval of 705.14–723.48 Mb on the Chinese Spring reference genome sequence v2.1 [24]. Previous studies reported that a series of formally designated *Pm* genes on chromosome 2BL were identified, including dominant genes *Pm6* [15], *Pm33* [18], *Pm51* [19], *Pm52* [11], *Pm63* [22] and *Pm64* [23] which indicated the chromosome 2BL is most likely to be an enrichment region for resistance genes.

*Pm6* was derived from *T. timopheevii* 2B/2G introgression and was moderate to highly susceptible to powdery mildew at the one-leaf stage to the two-leaf stage, but gradually increased resistance from the third leaf stage and reached complete resistance at the fourth leaf stage and later [16]. Wan et al. reported that *Pm6* was flanked by markers *CIT02g-18* and *CIT02g-20*, corresponding to the physical interval of 706.75–707.63 Mb and the candidate interval of *Pm6* had serious recombination suppression due to the introgression of the 2G chromosome segment. In contrast to those genes, *PmCG15–009* (705.14–723.48 Mb), derived from common breeding line ShiCG15–009, was highly resistant to powdery mildew from the first leaf stage to the whole stages shows no significant recombination suppression in our mapping population. Additionally, when tested with 57 co-segregated or closely linked markers of *Pm6*, only ten markers showed polymorphisms in ShiCG15–009, Yannong 21 and their derivative F_2:3_ families, which revealed a distinct genetic diversity between the candidate intervals of *PmCG15–009* and *Pm6*. In conclusion, *PmCG15–009* was significantly different from *Pm6*.

*Pm33* [18], a dominant powdery mildew resistance gene, was introduced from *Triticum carthlicum* accession PS5 and was mapped on the interval of 782.3–794.37 Mb. *Pm52* [20] was derived from the wheat cultivar Liangxing 99 and flanked by SSR markers *Xicssl326* and *Xicssl795*, referring to the physical interval of 589.14–594.63 Mb. In our study, the dominant gene *PmCG15–009* was delimited to an interval of 705.14–723.48 Mb on the Chinese Spring reference genome sequence v2.1, which was significantly different from *Pm33* and *Pm52* based on the physical interval and/or origins.

*Pm51* [19], *Pm63* [22] and *Pm64* [23] were derived from *T. ponticum*, Iranian wheat landrace PI 628024, and wild emmer, respectively. Although the physical interval of *PmCG15–009* overlapped that of *Pm51* (718.30–747.73 Mb), *Pm63* (718.80–731.82 Mb,) and *Pm64* (703.85–718.80 Mb), their source was different from each other. More importantly, all the closely linked markers or co-segregated markers of these three genes, including six for *Pm51*, six for *Pm63* and ten for *Pm64*, were not polymorphic between resistant parent ShiCG15–009 and susceptible parent Yannong 21 and two bulks, which indicated a various genetic diversity between the candidate intervals of *PmCG15–009* and these of the tested genes. Taken together, *PmCG15–009* was different from those documented genes on chromosome 2BL, which might be a novel gene or allele. To further provide more reliable evidence for their relationship, allelism tests and cloning of these genes are necessary in the future to further provide more reliable evidence for their relationship.

So far, 11 race-specific *Pm* genes have been cloned successively. Among them, *Pm3* [25], *Pm8* [26], *Pm2* [27], *Pm17* [6], *Pm60* [28], *Pm21* [29, 30], *Pm5e* [31], *Pm41* [32] and *Pm1a* [33] encoded coiled-coil nucleotide-binding site leucine-rich repeat protein (CC-NBS-LRR). *Pm4* [34] and *Pm24* [35] encoded a putative serine/threonine kinase and tandem kinase protein (TKP) with putative kinase-pseudokinase domains, respectively. In plants, NLR proteins and protein kinases are the major classes of disease resistance genes. NLR functions as intracellular immune receptor that recognizes pathogen effectors and activates effector-triggered immunity (ETI) and protein kinases are important for transmembrane signaling that regulates plant development and adaptation to diverse environmental conditions [36, 37]. In the candidate interval of *PmCG15–009*, 194 high confidence genes were annotated based on the IWGSC Chinese Spring reference genome v2.1, and only 14 genes are associated with disease resistance. Furtherly, qRT-PCR analysis showed that the transcript levels of six genes were induced at different degree by the *Bgt* isolate E09 between the resistant parent ShiCG15–009 and susceptible parent Yannong 21. Notably, the gene *TraesCS2B03G1283800*, encoding a serine threonine-protein kinase, showed high expression in ShiCG15–009 at 4 hpi following *Bgt* inoculation. *TraesCS2B03G1276100* and *TraesCS2B03G1266900* were significantly upregulated in resistant parent ShiCG15–009 but not changed in susceptible Yannong 21. Considering the expression patterns, these genes could be the candidate gene of *PmCG15–009* or regulatory genes involved in the resistance process. These data provide a significant direction at dissecting the resistance pathways. Of course, further studies are needed to investigate whether these genes are candidate genes of *PmCG15–009*.

When a novel gene was discovered, the rational utilization was the next challenge in wheat breeding programs. The elite wheat breeding line ShiCG15–009 showed not only highly resistance to powdery mildew at all the stages but excellent agronomic traits, thus should be a valuable resource for genetic research and wheat resistance improvement. To accelerate the transfer of *PmCG15–009* in MAS, we evaluated the availability of 20 markers linked or co-segregated with *PmCG15–009* in 46 susceptible commercial cultivars/lines. The results showed that 18 of 20 markers could be used singly or in combination in MAS for tracking *PmCG15–009* in the background of those susceptible cultivars. Also, we have made many hybrid combinations between ShiCG15–009 and several susceptible commercial wheat cultivars and obtained the BC_1_F_2_ and F_3_ segregation populations. In future, *PmCG15–009* will play an important role in wheat breeding programs.

## Conclusion

In the present study, a dominant powdery mildew resistance gene *PmCG15–009* was identified in wheat breeding line ShiCG15–00 and located within 705.14–723.48 Mb on chromosome 2BL. Based on the physical position, origin and genetic diversity, *PmCG15–009* is most likely a novel *Pm* gene. 18 molecular markers available for marker-assisted selection were selected for tracking *PmCG15–009* in breeding. Our study can be valuable for theoretical research and wheat breeding application.

## Materials and methods

### Plant materials and pathogen isolates

Wheat breeding line ShiCG15–009, released from Hebei Province, was highly resistant to powdery mildew at the adult and seedling stages, whereas wheat cultivar Yannong 21 was highly susceptible. The F_1_, F_2,_ and F_2:3_ populations, derived from the cross of ShiCG15–009 and Yannong 21, were used to map the powdery mildew resistance gene(s) in ShiCG15–009. Wheat cultivar Mingxian 169 which didn’t carry any known *Pm* gene was used as the susceptible control for phenotypic identification and served as the *Bgt* inoculum spreader. The *Bgt* isolate E09, collected from Beijing city in 1993 and currently prevalent in the main wheat producing regions of China, which was virulent to powdery mildew resistance gene *Pm6* and avirulent to *Pm33*, *Pm51*, *Pm52*, *Pm63* and *Pm64* on the chromosome arm 2BL [38], was used to evaluate the mapping populations. Prevalent powdery mildew *Bgt* isolates E20 and E31 with broad virulent spectrum were also used to test the wheat breeding line ShiCG15–009 (Table S1).

### Reactions to powdery mildew at the seedling stage

Resistance evaluation to powdery mildew was carried out in a greenhouse. Seedlings were grown in rectangular trays (54 × 28 × 4.2 cm), each tray had 128 cells (3.2 × 3.2 × 4.2 cm) and the susceptible check Mingxian 169 was planted with three cells randomly in the trays. For the F_2:3_ families derived from the cross ShiCG15–009 and Yannong 21, each of the families was tested with at least 20 seeds to confirm the genotype of the F_2_ plants. At the one-leaf stage, all seedlings were inoculated with fresh conidiospores increased on Mingxian 169 seedlings and incubated at a greenhouse with a daily cycle of 14 h of light at 22 °C and 10 h of darkness at 18 °C. 10–14 days later, when the spores were fully developed on the first leave of susceptible control Mingxian 169, infection types (ITs) on each plant were assessed on a 0–4 scale, of which 0 = no visible symptoms and signs, 0; = necrotic flecks without sporulation, 1 = sparse aerial hypha and little sporulation, the diameter of colonies less than 1 mm, 2 = moderate aerial hypha and sporulation, diameter of colonies less than 1 mm, 3 = thick aerial hypha and abundant sporulation, diameter of colonies more than 1 mm, and 4 = abundant sporulation with more than 80% of the leaf area covered with aerial hypha, with IT 0, 0;, 1 and 2 being regarded as resistant, and IT 3 and 4 as susceptible [39]. All tests were repeated three times to assure the reliability of the data.

### Marker analysis

Total genomic DNA was extracted from young leaf tissues following a procedure described by Sharp et al. (1988) [40]. The resistant and susceptible DNA bulks which consisted of 20 homozygous resistant and 20 homozygous F_2:3_ families of ShiCG15–009 and Yannong 21 were used in DNA-based Bulked Segregant Analysis (BSA) to validate polymorphic markers [41].

Three hundred and twenty one molecular markers evenly distributed across all the chromosomes [42–47] were selected for an initial survey of polymorphism between resistant and susceptible parents and bulks. Then, the polymorphic markers between the parents and the bulks were used to genotype the F_2:3_ families of ShiCG15–009 and Yannong 21 for mapping of the *Pm* gene(s) in ShiCG15–009. In addition, 200 markers based on the simple sequence repeat (SSR) in the target region on chromosome 2BL were designed and were also used to genotype the F_2:3_ families. The corresponding genomic sequences of *PmCG15–009* target region were used as templates to search SSR with the software SSR Hunter, and the parameters as follows: the number of nucleotide repeat units is one to six bp and the number of repeats is more than five. The SSR markers were designed with Primer 5 software. Polymorphism of SSR markers were examined using the parents and the contrasting DNA bulks.

PCR amplification was performed with a 10 μl volume which contained 5 μl 2 × Taq Master Mix (Vazyme, China), 1 μl 50 ng/μl template DNA and 0.5 μl 10 μM/μl primers. The PCR amplification conditions were as follows: pre-denaturation at 94 °C for 5 min followed by 36 cycles of 94 °C for 30 s, 50 to 65 °C (depending on the specific primers) for 40 s, 72 °C for 40 s to 120 s (depending on the target bands), finally extension at 72 °C for 10 min and preservation at 25 °C. PCR products were separated in 8% non-denaturing polyacrylamide gels with a 29:1 ratio of acrylamide and bis-acrylamide, then silver stained and visualized as previously described [48].

### Statistical analysis

After obtaining phenotypic data and the genotypic data of the F_2:3_ families derived from the cross ShiCG15–009 and Yannong 21, Chi-squared (χ^2^) tests for goodness-of-fit were used to evaluate deviations of observed data from expected segregation ratios. The software MAPMAKER/Exp (version 3.0b) was used to determine linkage with a LOD score of 3.0 as the threshold for declaration of linkage [49]. Genetic distances were estimated from recombination values using the Kosambi mapping function [50].

### Genetic diversity comparison with the documented *pm* genes on the chromosome 2BL

To investigate the genetic diversity of the candidate interval of *Pm* gene(s) in ShiCG15–009 and the known *Pm* genes on chromosome 2BL. 99 markers closely linked to those *Pm* genes were tested for polymorphisms between resistant parent ShiCG15–009 and susceptible parent Yannong 21 and their derived resistant and susceptible bulks.

### Prediction and analysis of candidate genes

The flanked markers were aligned to Chinese Spring reference genome sequence v2.1 to obtain the corresponding physical interval of the candidate gene(s) in ShiCG15–009. Then, the annotated disease resistance genes within the mapped interval were used to analyze the expression patterns between resistant parent ShiCG15–009 and susceptible parent Yannong 21 after inoculating with the *Bgt* isolate E09 at different times.

Total RNA of ShiCG15–009 and Yannong 21 were extracted from leaves after inoculating *Bgt* isolate E09 at 0, 0.5, 2, 4, 12, 24, 36 and 48 hpi using TRIzol reagent (Invitrogen, USA). About 2 μg of RNA was used for reverse transcription with a FastQuant RT Kit (Tiangen, China). The qRT-PCR assays were performed using SYBR Premix Ex Taq (Takara, China) on the Bio-Rad CFX Connect real-time PCR system (BIO-RAD, USA). The expression pattern of each gene was calculated as a fold change using the comparative CT method [51]. For each sample, three technical replications were analyzed. The *TaActin* was used as the internal control for normalization. Primers used in this study were listed in Table S1.

### Evaluation of the markers for MAS

The 46 powdery mildew-susceptible wheat cultivars/lines from different major wheat producing regions and six reference cultivars/lines carrying known genes on the chromosome arm 2BL, including Coker747 (*Pm6*), Am9/3 (*Pm33*), CH7086 (*Pm51*), Liangxing 99 (*Pm52*), PI 628024 (*Pm63*) and WE35 (*Pm64*) were tested by using the flanked or co-segregated markers. If the polymorphic band(s) amplified by a marker were all same for ShiCG15–009 and the tested cultivars, this marker could not be used for MAS. However, the bands amplified in ShiCG15–009 were different from the tested cultivars, indicating that the marker was considered to be available for MAS in those genetic backgrounds.

## Supplementary Information

**Additional file 1: Table S1.** The virulence frequency of *Blumeria graminis* f. sp. *tritici* (*Bgt*) isolates E09, E31 and E20.

**Additional file 2: Fig. S1.** The original and unprocessed amplification patterns of *PmCG15-009*-linked markers *YTU103–101* in genotyping resistant parent ShiCG15–009, susceptible parent Yannong 21, and randomly selected F_2:3_ families of ShiCG15–009 × Yannong 21. **Fig. S2.** The original and unprocessed amplification patterns of *PmCG15–009*-linked markers *CIT02g–17* in genotyping resistant parent ShiCG15–009, susceptible parent Yannong 21, and randomly selected F_2:3_ families of ShiCG15–009 × Yannong 21. **Fig. S3.** The original and unprocessed amplification patterns of *PmCG15–009*-linked markers *CISSR02g-6* in ShiCG15–009, Yannong 21 and 15 wheat cultivars/lines susceptible to powdery mildew. **Fig. S4.** The original and unprocessed amplification patterns of *PmCG15–009*-linked markers *CIT02g-17* in ShiCG15–009, Yannong 21 and 15 wheat cultivars/lines susceptible to powdery mildew.

## Data Availability

All the data generated or analyzed during the current study were included in the manuscript. The raw data is available from the corresponding author on reasonable request.
