# Supplementary material for: Characterization and identification of the powdery mildew resistance gene in wheat breeding line ShiCG15–009
Source: BMC Plant Biol. 2023 Feb 23;23:113. doi: 10.1186/s12870-023-04132-y (PMC9948530; doi:10.1186/s12870-023-04132-y)
Supplement: Supplementary file 2 — Additional file 2: Fig. S1. The original and unprocessed amplification patterns of PmCG15-009-linked markers YTU103–101 in genotyping resistant parent ShiCG15–009, susceptible parent Yannong 21, and randomly selected F2:3 families of ShiCG15–009 × Yannong 21. Fig. S2. The original and unprocessed amplification patterns of PmCG15–009-linked markers CIT02g–17 in genotyping resistant parent ShiCG15–009, susceptible parent Yannong 21, and randomly selected F2:3 families of ShiCG15–009 × Yannong 21. Fig. S3. The original and unprocessed amplification patterns of PmCG15–009-linked markers CISSR02g-6 in ShiCG15–009, Yannong 21 and 15 wheat cultivars/lines susceptible to powdery mildew. Fig. S4. The original and unprocessed amplification patterns of PmCG15–009-linked markers CIT02g-17 in ShiCG15–009, Yannong 21 and 15 wheat cultivars/lines susceptible to powdery mildew. [file 12870_2023_4132_MOESM2_ESM.docx]

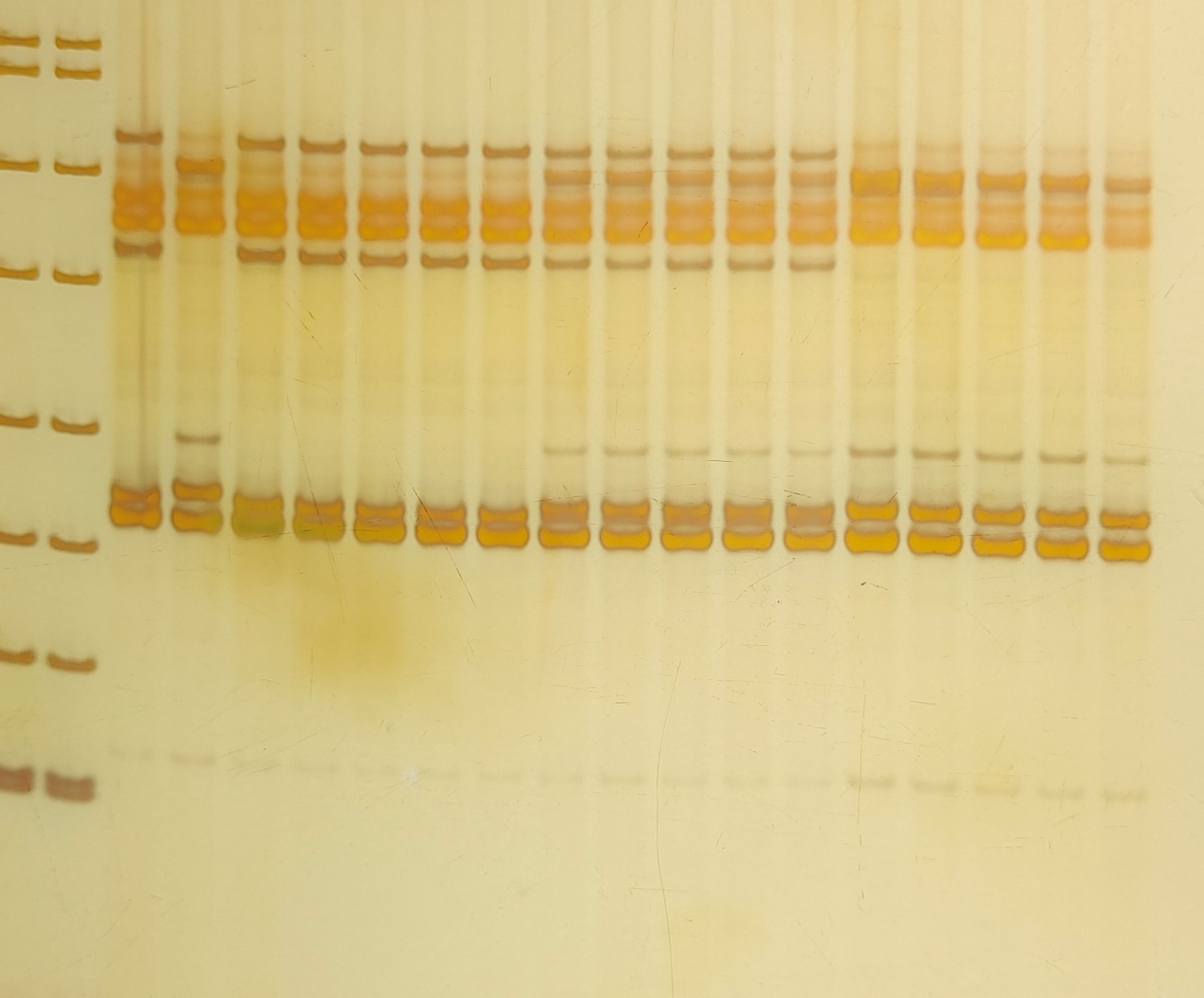


**Fig. S1** The original and unprocessed amplification patterns of *PmCG15-009*-linked markers *YTU103-101* in genotyping resistant parent ShiCG15-009, susceptible parent Yannong 21, and randomly selected F_2:3_ families of ShiCG15-009 ×Yannong 21.


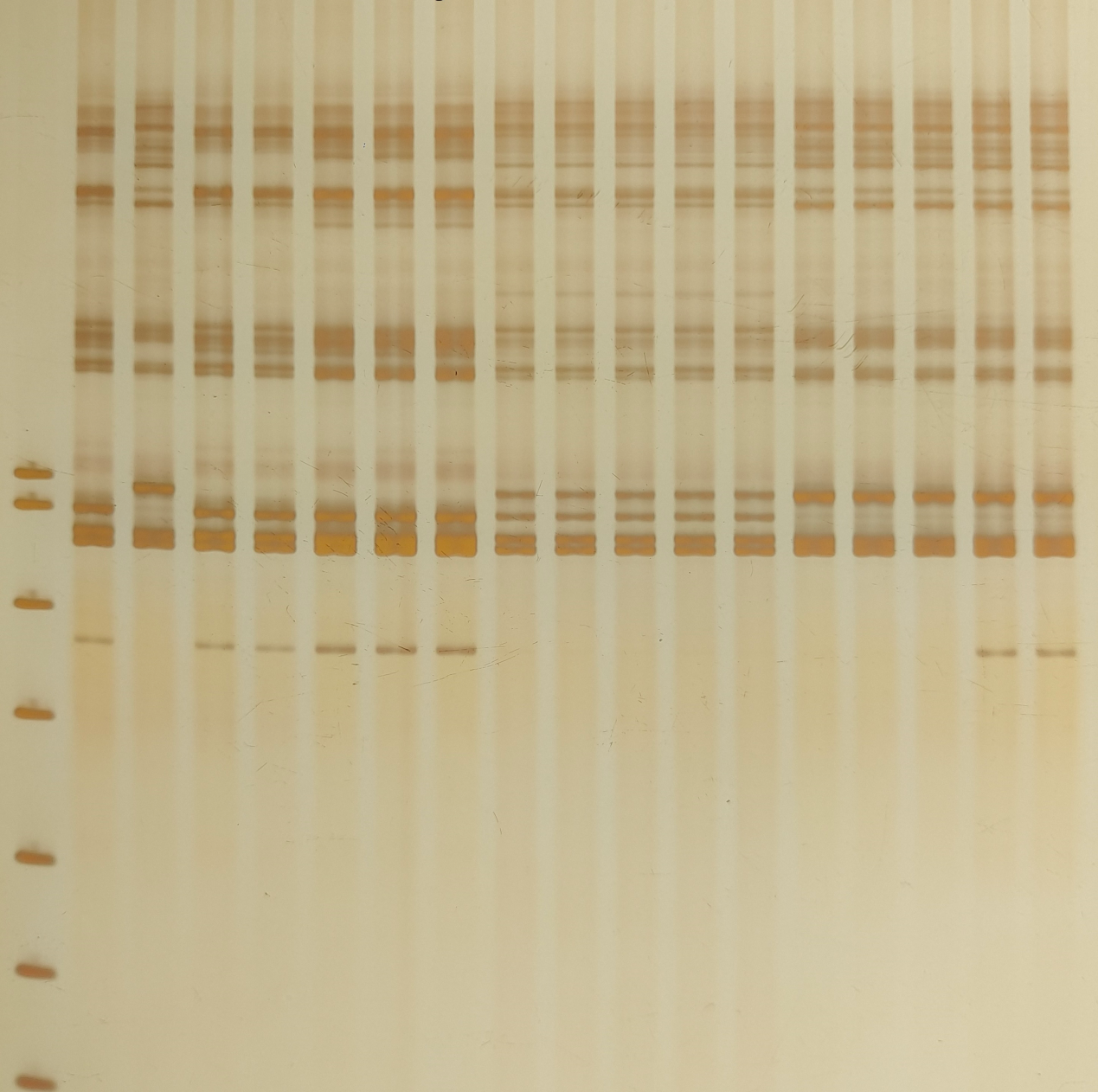


**Fig. S2** The original and unprocessed amplification patterns of *PmCG15-009*-linked markers *CIT02g–17* in genotyping resistant parent ShiCG15-009, susceptible parent Yannong 21, and randomly selected F_2:3_ families of ShiCG15-009 ×Yannong 21.


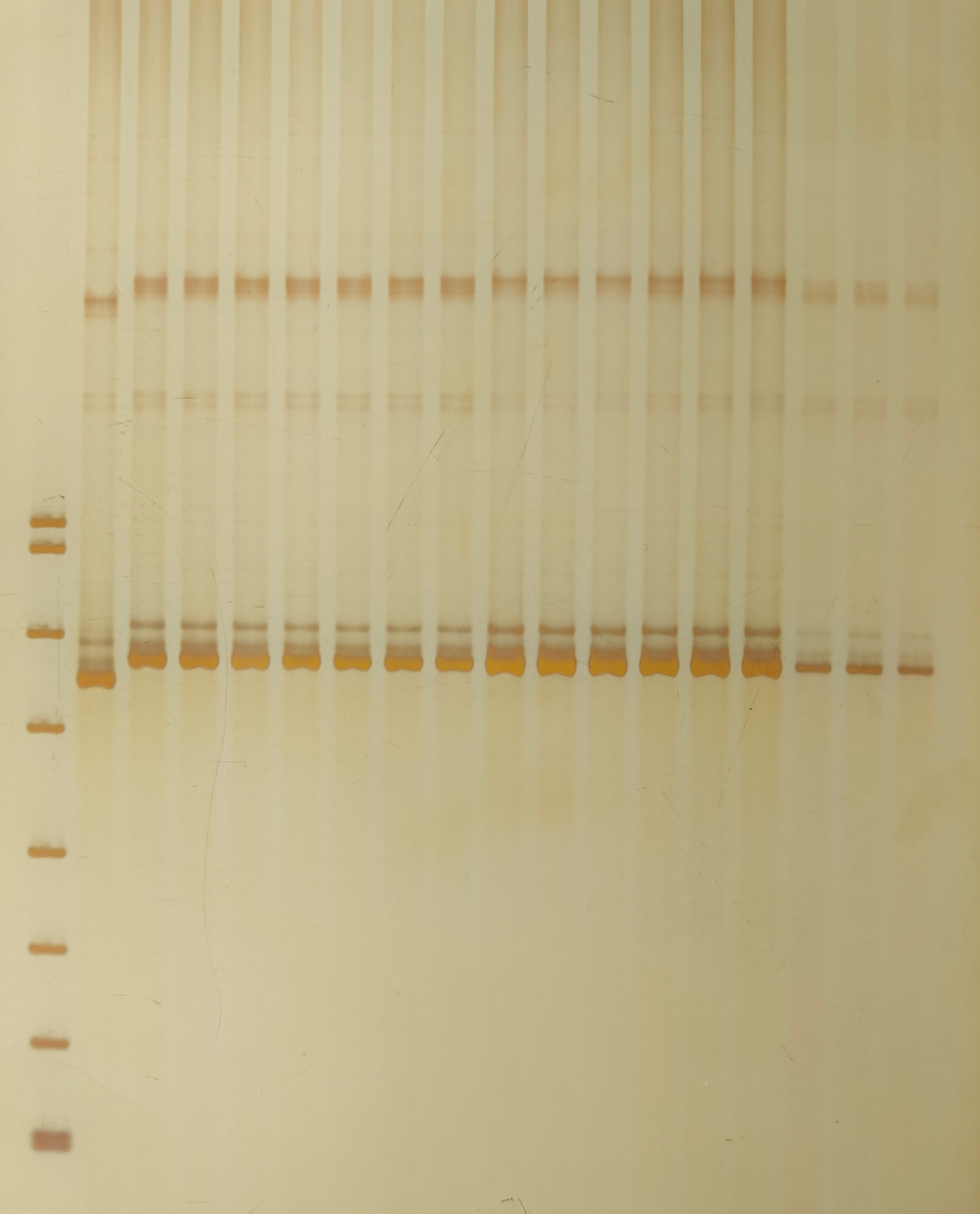


**Fig. S3** The original and unprocessed ampliﬁcation patterns of *PmCG15-009*-linked markers *CISSR02g-6* in ShiCG15-009, Yannong 21 and 15 wheat cultivars/lines susceptible to powdery mildew.


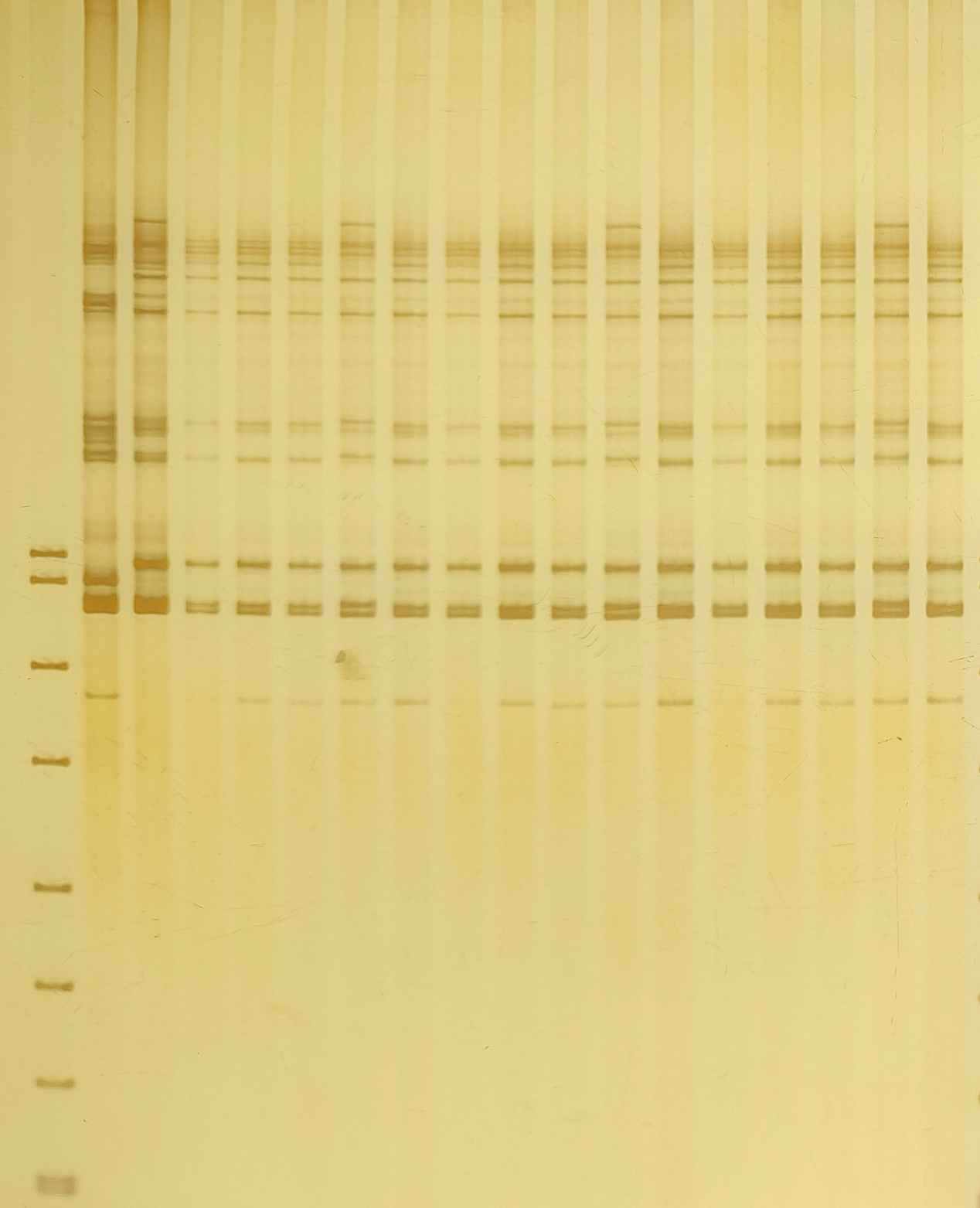


**Fig. S4** The original and unprocessed ampliﬁcation patterns of *PmCG15-009*-linked markers *CIT02g-17* in ShiCG15-009, Yannong 21 and 15 wheat cultivars/lines susceptible to powdery mildew.
